# Optimization of a Cefuroxime Axetil-Loaded Liquid Self-Nanoemulsifying Drug Delivery System: Enhanced Solubility, Dissolution and Caco-2 Cell Uptake

**DOI:** 10.3390/pharmaceutics14040772

**Published:** 2022-04-01

**Authors:** Arshad Ali Khan, Akhtar Atiya, Safia Akhtar, Yogesh Yadav, Kamal A. Qureshi, Mariusz Jaremko, Syed Mahmood

**Affiliations:** 1 The Robert M. Berne Cardiovascular Research Center, School of Medicine, University of Virginia, Charlottesville, VA 22903, USA; 2 Department of Pharmacognosy, College of Pharmacy, King Khalid University (KKU), Guraiger St., Abha 62529, Saudi Arabia; atkhan@kku.edu.sa; 3 Division of Endocrinology, School of Medicine, University of Virginia, Charlottesville, VA 22903, USA; safia.pharm@gmail.com (S.A.); dr.yogeshyadav2012@gmail.com (Y.Y.); 4 Department of Pharmaceutics, Unaizah College of Pharmacy, Qassim University, Unaizah 51911, Saudi Arabia; ka.qurishe@qu.edu.sa; 5 Smart-Health Initiative (SHI) and Red Sea Research Center (RSRC), Division of Biological and Environmental Sciences and Engineering (BESE), King Abdullah University of Science and Technology (KAUST), Thuwal 23955, Saudi Arabia; mariusz.jaremko@kaust.edu.sa; 6 Department of Pharmaceutical Technology, Faculty of Pharmacy, Universiti Malaya, Kuala Lumpur 50603, Malaysia

**Keywords:** cefuroxime axetil, pseudo-ternary phase diagrams, self-nanoemulsifying drug delivery system, Caco-2 cells, cytotoxicity, cellular uptake

## Abstract

Cefuroxime axetil (CA) is an oral cephalosporin which hydrolyzes rapidly to the active parent compound cefuroxime. CA is known to have incomplete oral bioavailability (30–50%) due to its poor solubility and enzymatic conversion to cefuroxime in the gut lumen. In order to overcome these drawbacks, a lipid-based self-nanoemulsifying drug delivery system (SNEDDS) has been developed and optimized. The SNEDDS formulations were prepared using the aqueous phase titration method. The greatest self-emulsifying area was found in the 2:1 Smix ratio. As a result, different SNEDDS formulations were carefully selected from this phase diagram based on their smaller droplet size < 100 nm, polydispersity index ≤ 0.5, dispersibility (Grade A), and transmittance (%) > 85%. Thermodynamic stability tests were carried out in order to rule out any metastable/unstable SNEDDS formulations. The droplet size, polydispersity index, zeta potential, and entrapment efficiency (% EE) of optimized CA-loaded SNEDDS (C-3) were 18.50 ± 1.83 nm, 0.064 ± 0.008, −22.12 ± 1.20 mV, and 97.62 ± 1.06%, respectively. In vitro release studies revealed that the SNEDDS formulation had increased CA solubility. CA-SNEDDS-C3 increased CA cellular uptake, possibly due to increased CA solubility and the inhibition of enzymatic conversion to cefuroxime. Finally, in terms of the improvement of oral bioavailability, CA-loaded-SNEDDS could be a viable alternative to commercially available CA formulations.

## 1. Introduction

Cefuroxime is a semi-synthetic cephalosporin class of antibacterial agent [1]. It is effective against Gram-positive and Gram-negative bacteria, allowing it to treat a wide variety of bacterial infections [2,3]. Unlike other second-generation cephalosporins, cefuroxime can penetrate the cerebrospinal fluid (CSF) [4,5].

Cefuroxime has a low oral bioavailability. Inserting a 1-acetoxyethyl ester group into the cefuroxime molecule yields the prodrug cefuroxime axetil (CA), which increases its lipophilicity and facilitates absorption through the intestinal mucosa [6]. After oral administration, the prodrug is rapidly hydrolyzed and de-esterified in the intestinal mucosa and portal circulation, releasing an active moiety of cefuroxime into the systemic circulation [7,8,9]. CA exhibits weak antibacterial activity in its prodrug form, and it only becomes active after de-esterification to cefuroxime [2]. CA is used to treat a variety of infections, but the determination of the best oral dosage schedule is difficult due to its poor and variable bioavailability. Moreover, coadministration with food could significantly improve the extent and absorption of CA bioavailability (36% to 52%) [2,10,11]. Furthermore, the rate and extent of bioavailability vary with the dosage form [12]. In addition, poor CA solubility and the enzymatic conversion to cefuroxime in the gut lumen are the main contributors to incomplete bioavailability. As a result, a formulation method that improves drug solubility while preventing enzymatic conversion to cefuroxime is highly desirable.

A lipid-based nanoformulation might be a good option for the improvement of the oral bioavailability of a lipophilic drug like CA. Among various lipid-based nanoformulations, self-nanoemulsifying drug delivery systems (SNEDDS) have demonstrated significant potential in the delivery of poorly aqueous-soluble lipophilic drugs to the systemic circulation [13,14]. SNEDDS are anhydrous, isotropic liquid mixtures of drugs, oil, surfactants, and/or cosurfactants that spontaneously form a nanoemulsion (20–200 nm) when diluted with water and gently agitated [15]. In the preparation of SNEDDS, drug molecules can be solubilized into dispersed oil droplets and encapsulated in the carrier system. However, SNEDDS require a higher amount of oil to incorporate drugs depending on their solubility. However, SNEDDS may tend to have stability issues such as an increase in globule size and drug precipitation during storage. As a result, the drug-loading capacity of SNEDDS, as well as the amounts of excipients used, limits its use. At high doses, surfactants and co-surfactants may irritate the gastrointestinal tract, which limits their daily uptake [16]. It is therefore necessary to formulate for the maximum drug loading capacity while using the lowest amount of solubilizing excipients [17]. SNEDDS improve lipophilic drug absorption by increasing their aqueous solubility and forming digestible, smaller droplets that can cross the intestinal barrier more easily [18,19].

The Caco-2 cell line, derived from a human colon carcinoma, was found to have several properties similar to small intestinal enterocytes [20,21,22]. They have been widely used as an in vitro model to study the intestinal permeability of a variety of drugs, including antibacterial cephalosporins [23,24,25]. They have also been shown to be effective models for the investigation of prodrug transport across mucosal barriers [26].

The objective of the present study was to develop and optimize a CA-loaded SNEDDS formulation with the aim of increasing the solubility and cellular uptake. The developed formulation could be a potential carrier for the oral delivery of CA.

## 2. Materials and Methods

### 2.1. Materials

The CA was gifted by Ranbaxy (Mohali, India). Tween 20^®^, ethyl oleate, eucalyptus oil, propylene glycol, polyethylene 400 (PEG 400), castor oil, dimethyl sulfoxide ≥99.5% (DMSO), thiazolyl blue tetrazolium bromide 98% (MTT) and methylcellulose were purchased from Sigma-Aldrich (St. Louis, MO, USA). Tween 80^®^, soybean oil and olive oil were purchased from R&M Chemicals Ltd. (Essex, UK). Capmul MCM EP was obtained from the Abitec Corporation (Janesville, WI, USA). Peceol and Maisine 35-1 were obtained from Gattefosse (Saint-Priest, France). Cremophor EL^®^ was obtained from BASF (Ludwigshafen, Germany). The dialysis membrane (MWCO 12,000 g/mole) was obtained from Sigma-Aldrich Sdn Bhd, Petaling Jaya, Malaysia. The Caco-2 cell line was bought from the American Type Culture Collection (ATCC) (Manassas, VA, USA). Dulbecco’s modified eagle’s medium (DMEM) was purchased from GE Healthcare Life Sciences (Logan, UT, USA). Trypsin 0.25% was purchased from GE Healthcare Life Sciences (Logan, UT, USA). The penicillin-streptomycin solution (100×) was obtained from Biowest (Nuaillé, France). Fetal bovine serum (FBS) and Dulbecco’s phosphate-buffered saline (DPBS) were purchased from Thermo Fisher Scientific (Waltham, MA, USA). The passive lysis buffer (5×) was purchased from Promega (Madison, WI, USA).

### 2.2. Methods

#### 2.2.1. HPLC Analysis

The HPLC method was used to determine the CA quantification for the solubility studies, in vitro dissolution studies, and cellular uptake studies. For the HPLC analysis, a Shimadzu chromatography system with an LC 20AD delivery pump (Kyoto, Japan) and a Phenomenex C18 column (250 mm length × 4.60 mm internal diameter) were used. At a flow rate of 1 mL/min and a column temperature of 30 °C, the mobile phase was made up of acetonitrile/ammonium phosphate buffer (0.025 M, pH 4.5) in a 25:75 (*v*/*v*) ratio. At a detection wavelength of 277 nm, 20 µL of the samples were injected into the HPLC system [1].

#### 2.2.2. Preparation of Blank SNEDDS

- Selection of the SNEDDS Components

The screening and selection of the excipients were carried out on the basis of the highest CA solubility using the equilibrium method. In a 5 mL centrifugation tube, an excess amount of CA (200 mg) was weighed and mixed separately with 1 mL of various oils, surfactants, and co-surfactants. The resultant mixture was vortexed for 2 min using a vortex mixer (Model ZX3, VELP Scientifica, New York, NY, USA). After that, the mixtures were placed in a reciprocating shaker water bath for 72 h at 37 ± 2 °C with 200 oscillations/min. After 72 h, the mixtures were centrifuged using a MiniSpin^®^ Eppendorf centrifuge (Hamburg, Germany) at 3000 rpm for 15 min to separate the undissolved CA. The resulting supernatant layer was filtered using a 0.45 m syringe filter (Whatman, New York, NY, USA). In order to determine the amount of CA in each excipient, a small amount of filtered supernatant (50 μL) was mixed with methanol (2 mL), and 20 μL of the sample was injected into the HPLC system.

An emulsification efficiency study was carried out to further screen the surfactant and co-surfactant in the selected oil. Different ratios (1:0, 1:1, 1:2, 2:1, 3:1, 4:1, and 5:1 (*v*/*v*)) of surfactant and co-surfactant (Smix) were added and mixed to form a homogeneous mixture in a 5 mL centrifugation tube. 10% *v*/*v* oil was mixed with 90% *v*/*v* Smix, and the mixture was equilibrated in a reciprocating shaker water bath at 25 ± 2 °C for 2 h. After the addition of distilled water, a nanoemulsion was formed. The resulting nanoemulsion was evaluated for its droplet size (DS), polydispersity index (PDI), and transmittance percentage (T%). A standard dilution was performed with distilled water in a 1:1000 ratio to determine the DS and PDI using a Zetasizer (Malvern Instrument, London, UK). On the other hand, a UV-Vis Spectrophotometer U-2000 (Hitachi, Yokohama, Japan) was used to determine the T% using a 10:1000 dilution of distilled water at 638.2 nm.

- Construction of the Phase Diagram

Pseudo-ternary phase diagrams were formed using the water titration method to prepare blank SNEDDS [27,28]. Briefly, a homogenous mixture of oil and Smix was titrated with distilled water at room temperature. The selected Smix ratio from the emulsification efficiency study was used for the phase diagram. Each Smix ratio was mixed with an increasing amount of oil (10–90%) and titrated with distilled water. Overall, the percentage of ternary phase (oil, surfactant, and co-surfactant) was maintained at 100%. Pseudo-ternary phase diagrams were constructed using a spontaneous emulsification method. In this method, the oil-Smix mixture was titrated by slowly adding water, and visual observations were taken based on its clarity [29]. Each phase diagram was constructed using self-nanoemulsifying zones that were clear, transparent, and easily flowable, with one axis representing water, the second oil, and the third Smix.

#### 2.2.3. Preparation of CA-Loaded SNEDDS

The phase diagram was used to select the SNEDDS formulations for the loading study. Almost the entire region’s SNEDDS zones were taken into account. For loading, 50 mg CA was dissolved in 1 mL of the selected oil, and vortexed for 30 s. The resulting oil mixture was mixed with the chosen Smix ratio. The obtained mixture was processed as a solubility study in a reciprocating shaker water bath for 72 h with 200 oscillations/min at 37 ± 2 °C. The obtained mixture was centrifuged using a MiniSpin^®^ Eppendorf centrifuge (Hamburg, Germany) for 15 min at 3000 rpm to remove any undissolved CA. The loading efficiency was calculated using Equation (1).

(1) $$\mathrm{Drug}\;\mathrm{Loading}\;\mathrm{Efficiency}\;(\%)=\frac{\mathrm{Amount}\;\mathrm{of}\;\mathrm{CA}\;\mathrm{in}\;\mathrm{the}\;\mathrm{formulation}\;\left(\mathrm{mg}\right)}{\mathrm{Total}\;\mathrm{amount}\;\mathrm{of}\;\mathrm{CA}\;\mathrm{added}\;\left(\mathrm{mg}\right)}\times 100$$

#### 2.2.4. Characterization of CA-Loaded SNEDDS

- Thermodynamic Stability and Self-Nanoemulsification Tests

Thermodynamic stability tests were performed on the developed CA-loaded SNEDDS in order to eliminate any unstable or metastable formulations. These tests included centrifugation, heating and cooling cycles, and freeze–thaw cycles [27]. The purpose of this study was to see how phase separation and temperature variations affected the stability of SNEDDS. In order to determine the stability of the selected formulations as an isotropic single-phase system, the test samples were diluted with distilled water in a 1:20 ratio and centrifuged using a MiniSpin^®^ Eppendorf centrifuge (Hamburg, Germany) at 3500× *g* rpm for 30 min. Following that, the rest of the formulations were subjected to three heating and cooling cycles, and incubated at 4 °C and 40 °C for 48 h; those that exhibited phase separation, creaming, or cracking were discarded. The formulations that remained stable at the heating and cooling cycles were then put through three freeze–thaw cycles at temperatures ranging from −20 to 25 ± 2 °C in a deep freezer for at least 48 h. The self-nanoemulsification test was conducted to watch for any phase separation or precipitation after diluting the sample with water, acid (0.1 N HCl), and phosphate buffer (pH 7.4). In brief, 1 mL CA-loaded SNEDDS was diluted 1:500 with water, acid buffer (0.1 N HCl), and phosphate buffer (pH 7.4), and was evaluated visually using the grading systems listed below.

Grade A: Clear nanoemulsion that forms quickly and spontaneously.
Grade B: Bluish, slightly less clear nanoemulsion that forms quickly and spontaneously.
Grade C: Turbid emulsion that forms slowly.
Grade D: Turbid emulsion that is dull and grayish.
Grade E: Turbid emulsion with visible oil globules on the surface.

- Physicochemical Characterization of the CA- SNEDDS

The selected CA-loaded SNEDDS were physicochemically characterized in order to determine their DS, PDI, zeta potential (ZP), T%, and surface morphology. The mean DS and PDI of the CA-SNEDDS were determined using a Zetasizer 1000 HSA (Malvern Instrument, London, UK). ZP was measured using a Zetasizer nanoseries Nano-Z (Malvern Instrument, UK). The T% of the CA-SNEDDS was determined using a UV-Vis Spectrophotometer U-2000 (Hitachi, Japan) at 638.2 nm. The dilution of the samples for DS, PDI, and T% analysis has already been mentioned. The surface morphology and structure of droplets of the optimized CA-SNEDDS formulation were evaluated using a Tecnai G2 20 Twin transmission electron microscope (FEI, Hillsboro, OR, USA). In order to make the slide, a drop of the formulation was applied to a 400-mesh copper grid and allowed to air dry. Then, it was negatively stained with phosphotungstic acid (2%) for 5 min at room temperature. The excess liquid was carefully removed with filter paper and allowed to dry before being observed.

#### 2.2.5. In Vitro Release Studies

The in vitro release of CA from SNEDDS was performed using a dialysis method in a USP-II dissolution apparatus (Santa Clara, CA, USA) with a rotation speed of 50 rpm at 37 ± 0.5 °C [30]. The CA-SNEDDS and CA-suspension (CA equivalent to the formulation) were reconstituted (1:20) and placed in an activated dialysis tube (MWCO 12,000 g/mole; Sigma, St. Louis, MO, USA) in a dissolution vessel containing 900 mL of simulated gastric fluid (SGF; pH 1.2) and simulated intestinal fluid (SIF; pH 6.8). Both SGF (1000 mL SGF contains 2 g NaCl, 7 mL HCl and distilled water q.s.; the pH was adjusted using 1 N NaCl) and SIF (1000 mL SIF contains 6.8 g KH2PO4, 77 mL 0.2 N NaOH and distilled water q.s.; the pH was adjusted to 6.8 using 0.2 N NaOH or 0.2 N HCl) media were prepared without enzymes. Then, 5 ml aliquots were removed from each vessel and replaced with the same volume of fresh dissolution media at intervals of 0, 5, 10, 15, 30 and 60 min. The CA was measured using the HPLC method at 277 nm after the samples were filtered through 0.45 μm membrane filters.

#### 2.2.6. Cell Culture Studies

Heterogeneous human epithelial colorectal adenocarcinoma (Caco-2) cells with passage numbers ranging from 20 to 21 were used for the study. The cells were grown at 37 °C in DMEM complete media supplemented with 10% *v*/*v* FBS and 1% *v*/*v* penicillin/streptomycin in a humidified 95% air/5% CO_2_ atmosphere.

- Cytotoxicity Study

The cytotoxicity study was conducted using an MTT assay to evaluate the toxicity of the blank SNEDDS formulation. The assay was carried out using the previously described method [31]. Briefly, cells were seeded at a density of 5 × 104 cells per well in a 96-well plate. In total, 200 μL of the culture media were distributed to each well. The cells were incubated with varying percentages—0 (control), 0.05, 0.1, 0.25 and 0.5 percent *v*/*v*—of blank SNEDDS formulation diluted with culture media for 24 h at 37 °C. DMEM was used as a control. After 24 h, 30 μL 5 mg/mL MTT solution was added to each well and incubated for another 4 h. The supernatant layer was removed after 4 h of incubation, and 200 μL DMSO was added to each well, followed by 5 min of gentle rotation on an orbital shaker. The absorbance of each well was measured at 560 nm using a MultiskanTM FC microplate reader (Thermo Waltham, Waltham, MA, USA). The percentage of viable cells was calculated using the following Equation (2):

(2) $$\mathrm{Cell}\;\mathrm{viability}\;(\%)=\frac{\mathrm{Abs}\;\left(\mathrm{test}\right)-\mathrm{Abs}\;\left(\mathrm{blank}\right)}{\mathrm{Abs}\;\left(\mathrm{control}\right)-\mathrm{Abs}\;\left(\mathrm{blank}\right)}\times 100$$

where Abs (test) represents the absorbance of cells treated with blank SNEDDS, Abs (control) represents the absorbance of cells treated with DMEM alone, and Abs (blank) represents the absorbance of blank cells (DMEM).

- Cellular Uptake Study

For the uptake study, three different dilutions of the optimized CA-SNEDDS formulation (without antibiotics and serum) were prepared in DMEM media. Similar dilutions of the CA suspension were prepared as a control [32]. Caco-2 cells were seeded in 48-well plates at a density of 50,000 cells per well with DMEM complete media until they reached >85% confluence and formed a monolayer. CA-SNEDDS and CA suspension containing 0.5 mM of CA were then used to replace the DMEM complete media, and the 48-well plates were incubated at 37 °C for 4 h. The test samples were pipetted out of each well after the incubation period, and the Caco-2 monolayer was washed three times in DPBS to remove any remaining test samples or dead cells. In order to extract the absorbed CA in Caco-2 cells, 0.2 mL passive lysis buffer and 0.2 mL methanol were added to each well. The lysed Caco-2 cells were separated by transferring the mixtures into centrifuge tubes and centrifuging them at 14,000× *g* rpm for 10 min using a MiniSpin^®^ Eppendorf centrifuge (Hamburg, Germany). The concentration of CA in the supernatants was determined using HPLC.

#### 2.2.7. Statistical Analysis

At the very least, each experiment was carried out in triplicate. The data were analyzed using a one-way analysis of variance (ANOVA), followed by Tukey-HSD post-hoc analysis. IBM^®^ SPSS^®^ Statistical software was used for the statistical analysis (Version 22, New York, NY, USA). The mean and standard deviation (mean SD) were used to express all of the data. The difference is statistically significant when *p* < 0.05.

## 3. Results and Discussion

### 3.1. Solubility Studies, Excipient Screening and Selection

**Figure 1:**
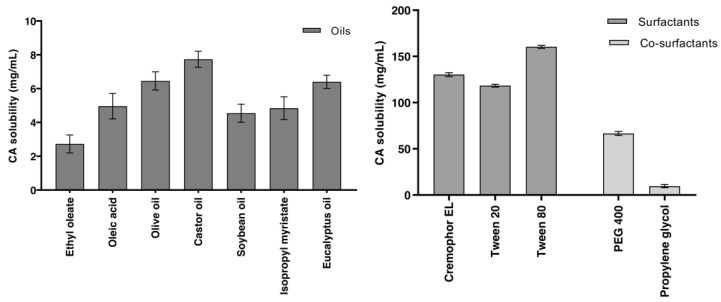
The solubility of CA in various excipients (*n* = 3, mean ± SD).

As previously stated, the solubility of CA molecules in various components of SNEDDS—such as oils, surfactants and co-surfactants—is the primary criterion for their selection (Figure 1). The increased solubility of the drug in the oil phase was essential to maintain the drug in the solubilized state and prevent drug precipitation in the gut lumen during dilution. Higher drug solubility in oil means less oil in the formulation, which means that less of the surfactants and co-surfactants are needed to emulsify drug-loaded oil droplets [30]. Figure 1 shows that CA solubility in different excipients. Among the various oils, castor oil (7.74 ± 0.47 mg/mL) had the highest CA solubility. In the surfactant screening, nonionic surfactant was considered over ionic surfactant for oral administration [33]. In addition, it can also be helpful in the preparation of a stable nanoemulsion over a range of pH and ionic strengths, and it improves the intestinal absorption of the loaded drug [34]. Among the screening surfactants, Tween 80 and Cremophor EL, which had the highest CA solubilization capacity (160.33 ± 1.53 mg/mL and 130.52 ± 2.08 mg/mL, respectively), were chosen for further evaluations. The addition of the co-surfactant in the SNEDDS component increases the nanoemulsion region, and hence the emulsification efficiency. PEG 400 was selected as a co-surfactant due to its higher CA solubility (66.58 ± 2.12 mg/mL).

It is not reasonable to select a surfactant solely on the basis of CA solubility. However, it is possible that a surfactant with a high solubilizing potential does not have a high affinity for the selected oil. As a result, the surfactant and co-surfactant were chosen for their ability to emulsify oil, rather than just their ability to solubilize CA. Following the solubility study, emulsification studies were carried out to assess the emulsifying ability of the selected surfactant and co-surfactant. As stated in Section 2.2, 10% *v*/*v* of the selected castor oil was mixed with varying Smix ratios (90% *v*/*v*). In this test, Tween 80 and cremophor EL were mixed separately with PEG 400 to form two different Smix (Tween 80: PEG 400 and cremophor EL: PEG 400). A fixed oil ratio (10% *v*/*v*) could be beneficial in the production of spontaneous and self-emulsifying nanosize globules (Pouton, 2000). Smix ratios that produced DS < 150 nm, PDI < 0.7, and T% > 85 in the selected oil were chosen for further investigation. After considering these criteria, the Smix with the Tween 80:PEG 400 combination showed a low emulsifying ability with castor oil, and formed a turbid solution (T% < 85), which was eliminated from further investigation, while the Smix with the cremophor EL:PEG 400 combination was continued for the construction of the phase diagram.

### 3.2. Construction of the Pseudo-Ternary Phase Diagrams and Drug Loading

**Figure 2:**
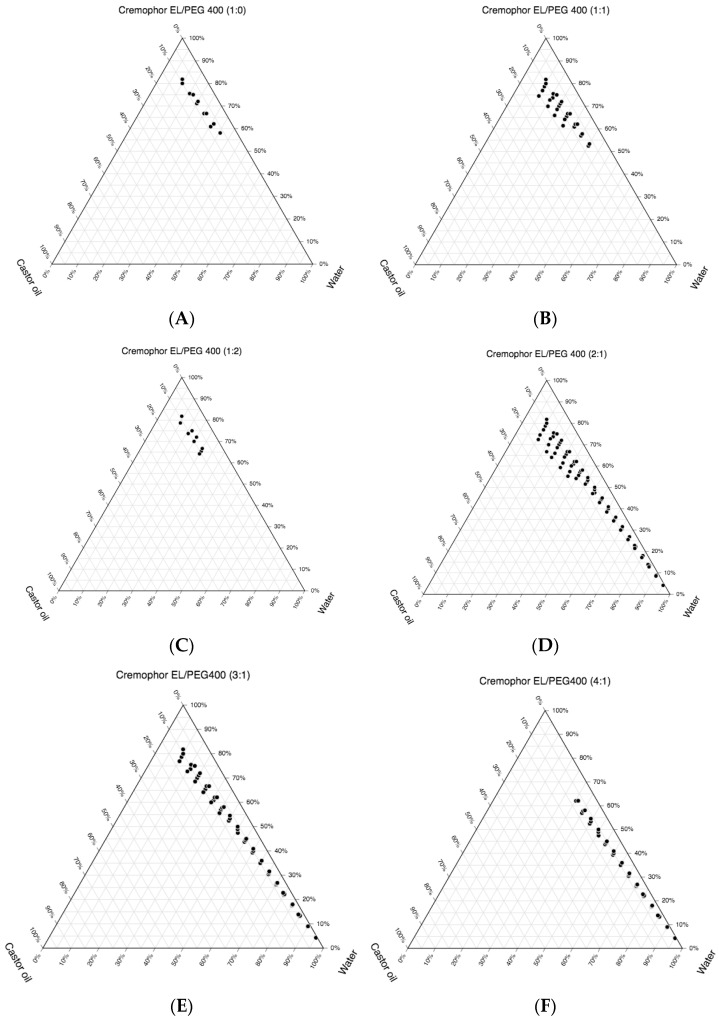
Pseudo-ternary phase diagrams were constructed using the aqueous titration method for the SNEDDS zones (dotted region) for castor oil, cremophor EL, PEG400 and water at Smix ratios of (**A**) 1:0, (**B**) 1:1, (**C**) 1:2, (**D**) 2:1, (**E**) 3:1 and (**F**) 4:1.

For the development of the CA-SNEDDS, pseudo-ternary phase diagrams were constructed using castor oil, Cremophor EL, PEG 400 and water. Figure 2 shows that the Smix ratio of 1:0 (with no co-surfactant) had a poor self-nanoemulsifying area (Figure 2A). The maximum amount of castor oil emulsified by a 1:0 ratio was 10% *v*/*v*, compared to the 80% *v*/*v* of Smix. However, when the amount of cosurfactant (PEG 400) in the Smix ratio was increased from 1:0 to 1:1, the self-nanoemulsifying area increased (Figure 2B). The maximum amount of castor oil that was emulsified by a 1:1 ratio was higher than that in 1:0 of the Smix ratio, i.e., 15.44% *v*/*v* with respect to 74.51% *v*/*v* Smix (Table 1). When the PEG 400 concentration was increased in the Smix ratio from 1:1 to 1:2 while the cremophor EL concentration was kept constant, the self-nanoemulsifying area did not seem to increase further. The maximum amount of castor oil (11.24% *v*/*v*) emulsified by a 1:2 Smix ratio (78.65% *v*/*v*) was found to be the same as that of a 1:1 Smix ratio (Figure 2C). When the ratio of the surfactant increased in the Smix ratio from 1:0 to 2:1 (Figure 2D), the self-nanoemulsifying area increased significantly in comparison to previous Smix ratios (1:0, 1:1 and 1:2). The maximum amount of castor oil was emulsified by the 2:1 ratio was observed to be 17.21% *v*/*v* with respect to 72.36% of the Smix (Table 1). However, by further increasing the amount of surfactant in the Smix ratio (3:1), the self-nanoemulsifying areas were found to be reduced in comparison to the 2:1 Smix ratio (Figure 2E). The maximum amount of castor oil that was emulsified by the 3:1 Smix ratio was observed to be 12.82% *v*/*v* with respect to 76.92% *v*/*v* (Table 1). When the amount of cremophor EL was further increased with respect to PEG 400 (Smix ratio of 4:1), the self-nanoemulsifying area was observed to be decreased further compared to the 1:1, 2:1 and 3:1 (Figure 2F). The maximum amount of castor oil that was emulsified by the 4:1 ratio was recorded as 8.13% *v*/*v* by incorporating around 56.91% *v*/*v* of the Smix (Table 1). According to the aqueous phase titration method, the greatest self-emulsifying area was found in the 2:1 Smix ratio (Figure 2D). As a result, different SNEDDS formulations for CA loading were carefully chosen from Figure 2D. In the selection of the formulations (C1–C10), the castor oil concentration was maintained at more than 5% *v*/*v*, while the Smix concentration was maintained at less than 50% *v*/*v*. A summary of the findings from the pseudo-ternary phase diagram are mentioned in Table 2.

### 3.3. Characterization of the CA-Loaded SNEDDS

- Thermodynamic Stability Study

The primary aim of the thermodynamic stability tests was to eliminate any metastable/unstable SNEDDS because, during phase titration studies, the whole observation was made visually. As a result, several thermodynamic stability studies were performed on selected CA-SNEDDS formulations. Table 3 summarizes the qualitative findings of these investigations. It shows that all of the developed CA-SNEDDS formulations (C1–C10) passed all of the steps of the thermodynamic stability tests. The CA-SNEDDS formulations (C1–C10) were then subjected to a self-nanoemulsification efficiency test, which is required for oral-emulsifying formulations [29]. The main objective of this analysis was to examine whether drug precipitation or phase separation occurred after gentle agitation with water, acid (0.1 N HCl), and phosphate buffer (pH 6.8); the qualitative results are shown in Table 3 [29,35]. In the presence of all three diluents, all of the CA-SNEDDS (C1–C10) passed with grade A. Overall, these findings show that the CA remained in a solubilized state at the molecular level in the developed SNEDDS, and that the self-nanoemulsification behavior of all of the formulations was pH independent [28].

- Physicochemical Characterization of the CA-SNEDDS

The physicochemical characterization of the developed CA-SNEDDS formulations (C1–C10) is shown in Table 4. In this study, the mean DS, PDI, ZP and T% were investigated. Table 4 shows that the mean DS of CA-loaded SNEDDS (C1–C10) ranges between 18.50 ± 1.83 and 33.43 ± 0.83 nm. Increasing the Smix (% *v*/*v*) reduces the droplet size significantly. The mean DS varied slightly when the castor oil concentration was kept constant at 5% *v*/*v,* and the concentration of Smix varied from 30 to 45% *v*/*v* (C1–C4). With an increasing Smix concentration, the mean DS reduces from 26.17 ± 2.05 to 18.50 ± 1.83 nm (Table 1 and Table 4). In C5 and C8, the Smix concentrations were 56.91 and 55.17% *v*/*v*, respectively, while the oil phase (castor oil) concentration increased from 8.13 to 13.79% *v*/*v*, resulting in a considerable increase in the mean DS from 23.80 ± 1.45 to 33.43 ± 0.83 (Table 1 and Table 4). The mean DS of these formulations was found to increase rapidly as the castor oil concentration in the SNEDDS increased (Table 4). These findings showed that the Smix had a minor impact on the mean DS, whereas castor oil had a larger impact. Overall, formulation C8 had the largest droplet size (33.43 ± 0.83 nm), which was most likely due to the presence of the highest amount of castor oil (13.79 percent *v*/*v*). Formulation C3 had the smallest droplet size (18.50 ± 1.83 nm), which was most likely due to the presence of the lowest amount of castor oil (5% *v*/*v*).

The PDI can be used to assess the uniformity of the droplets in an SNEDDS that has been developed. Table 4 shows that the PDI of formulations C1–C10 ranged from 0.064 to 0.170. Formulation C3 had the lowest PDI (0.064), indicating that the droplet size distribution was more uniform than those of the other formulations. Formulation C8, on the other hand, had the highest PDI value (0.170). In all of the formulations (C1–C10), the lower PDI value (<0.2) indicates a uniform droplet size distribution.

The ZP must be measured in order to determine the net surface charge and stability of the developed SNEDDS. The ZP values for the formulations (C1–C10) ranged from −22.12 ± 1.20 to −12.35 ± 2.06 mV (Table 4). The lowest ZP value (−22.12 ± 1.20 mV) was found in formulation C3, while the highest ZP value (−12.35 ± 2.06 mV) was found in formulation C7. The presence of negatively charged fatty acid esters in castor oil may have contributed to the negative ZP values in all of the CA-SNEDDS. The higher the ZP (±30 mV), the stronger the repulsive forces between the nanoparticles, which prevent them from aggregating. Nanoparticles with a ZP of ±20 to ±40 have a lower tendency to aggregate or grow in particle size [36]. With ZP values of −22.12 ± 1.20 mV, formulation C3 was found to be the stable.

T% is a useful metric for the assessment of the transparent behavior of SNEDDS. Table 4 shows that the T% of the developed formulations (C1–C10) ranged between 96.23 and 98.32%. Formulation C3 had the highest T% value (98.32 ± 0.27%). On the other hand, formulation C7 had the lowest T% value (96.23 ± 0.15%). These results show that all of the CA-SNEDDS formulations (C1–C10) behaved transparently.

- Drug Loading Efficiency

For all of the selected formulations (C1–C10), the loading efficiency of CA was found to be in the range of 96.25 ± 1.22 to 99.41 ± 0.22, indicating uniform drug distribution in the formula (Table 4). The formulations C7, C9, and C10 were found to have the highest loading efficiency, with 99.41 ± 0.22%, 99.16 ± 0.48% and 99.04 ± 0.23%, respectively. This could be due to the higher concentration of Smix in these formulations, which has a greater capacity to solubilize CA. Furthermore, the higher oil concentration in these formulations may be responsible for the higher CA loading efficiency.

### 3.4. In Vitro Drug Release Studies

**Figure 3:**
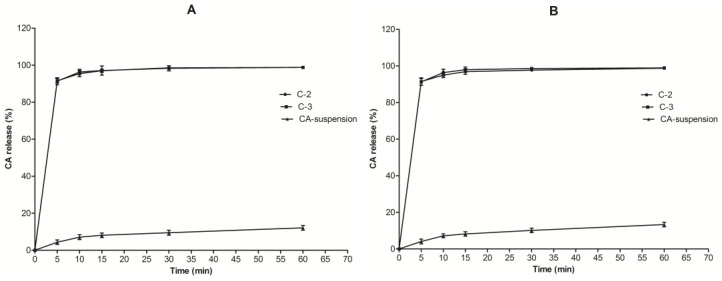
In vitro dissolution profile of the CA from CA-loaded SNEDDS and CA suspension: (**A**) CA (%) release in SGF (pH = 1.2); (**B**) CA (%) release in SIF (pH = 6.8). (*n* = 3, mean ± SD).

An in vitro release study was performed in order to compare the CA (%) release from the developed SNEDDS formulations (C2 and C3) and the CA suspension (containing the same amount of the drug). The release profile depicts the two-step process for SNEDDS, as shown in Figure 3A,B. The burst release phase, which can be attributed to the surface-associated drugs, was followed by a slower sustained release phase. The latter phase could be due to the diffusion-controlled dissolution of CA from the globules [37]. Other reasons for the burst release from the SNEDDS could be the smaller mean DS of both the formulation (the 21.23 ± 1.90 nm and 18.50 ± 1.83 nm sizes of C2 and C3, respectively) and ultimately the higher surface area, which permitted burst release. However, the initial CA release from the CA-suspension was found to be much slower than the optimized formulations C2 and C3. It was also found that optimized SNEDDS substantially enhanced the CA release, as more than 90% of the CA was released in the first 10 min of the study, compared to less than 5% from the CA suspension.

Moreover, the release pattern of CA for 60 min using six replicates in both formulations in both media (SGF and SIF) was more than 98% of the total amount of CA in the SNEDDS. In contrast, the release of CA (%) from the CA suspension was found to be less than 10% in both media (SGF and SIF) at the same time duration. As a result, the improved dissolution of CA from optimized SNEDDS may contribute to its higher in vivo absorption and bioavailability. The findings also backed up the hypothesis that nanosized emulsion droplets can improve the release of poorly soluble drugs [38]. Because both the C2 and C3 formulations showed a similar release pattern, the C3 formulation was selected for further study due to its lowest mean DS (18.50 ± 1.83), lowest PDI (0.064 ± 0.008), and highest ZP value (−22.12 ± 1.20 mV).

### 3.5. High-Resolution Transmission Electron Microscope (HRTEM)

**Figure 4:**
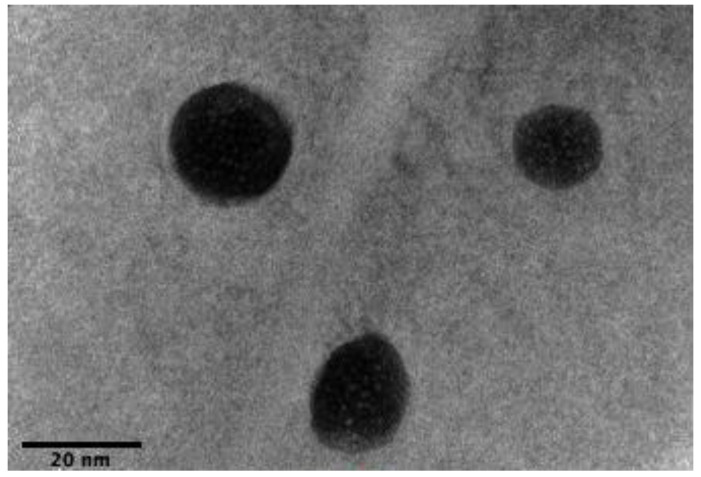
HRTEM image of CA-SNEDDS-C3 at a magnification of 220,000×.

HRTEM images of formulation C3 were taken and evaluated for its surface morphology and droplet size (Figure 4). All of the C3 droplets were found to be nanometer-sized and spherical in shape.

### 3.6. Cell Culture

- Cytotoxicity Assay

Caco-2 cells are commonly used to predict intestinal absorption in vitro. However, the toxicity of the prepared formulations can artificially increase permeation by damaging cell monolayers, resulting in unreliable results. In order to ensure that this did not happen, the cytotoxicity studies were performed prior to the cellular uptake studies. Though the excipients used in the preparation of SNEDDS are considered safe for oral administration, the cytotoxicity of their combination must be evaluated.

**Figure 5:**
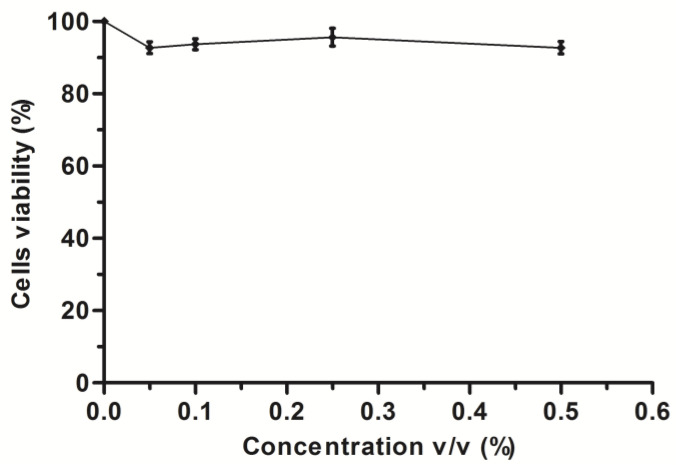
The cytotoxicity profile of different concentrations *v*/*v* (%) of blank SNEDDS formulations (*n* = 3, mean ± SD).

For the evaluation of cytotoxicity, we chose the commonly used method named the MTT cell viability assay (Figure 5). This cytotoxicity assay was developed by Mossman, and it is now one of the most widely used methods for the determination of cytotoxicity [39]. The MTT assay involves the conversion of a water-soluble thiazolyl blue tetrazolium bromide dye MTT (3-(4.5-dimethyl- thiazol-2-yl)-2.5-diphenyltetrazolium bromide) into an insoluble purple-colored formazan by cellular NADH. A spectrophotometer is then used to measure the absorbance of the solubilized formazan solution [40]. The MTT assay is a sensitive assay, with optical density readings reflecting metabolic activities [41]. The assay detects living cells but not dead ones, and the signal produced is proportional to the degree of cell activation [39]. After a 4-h incubation period, the MTT assay revealed that the blank SNEDDS had low cytotoxicity, and induced no toxic effects. The cell viability of the blank SNEDDS formulation was found to be greater than 90%.

- Cellular Uptake Study

The insufficient oral bioavailability of CA (less than 50%) is thought to be due to esterase hydrolysis and the p-glycoprotein efflux pump (P-gp). Previous investigation found that intestinal esterases hydrolyzed CA to the poorly absorbed parent drug cefuroxime prior to absorption, and lower the oral bioavailability of CA in vivo [12,42]. Similarly, it was also observed that CA rapidly hydrolyzed intracellularly after Caco-2 cellular uptake, and could not detected within the cells [24]. Moreover, P-gp is found in abundance in the epithelium of the small intestine, and is believed to limit transcellular drug absorption [43]. As a result, it was hypothesized that P-gp-mediated efflux could play a significant role in lowering the oral bioavailability of CA. Because Caco-2 cells have been shown to have P-gp-like activity, they have been used to investigate CA absorption in the presence and absence of verapamil, a well-known inhibitor of P-gp-mediated efflux. Barrett and colleagues strongly proved, in their study, that CA is a substrate for P-gp-mediated efflux, which may contribute to the insufficient bioavailability of CA [1].

**Figure 6:**
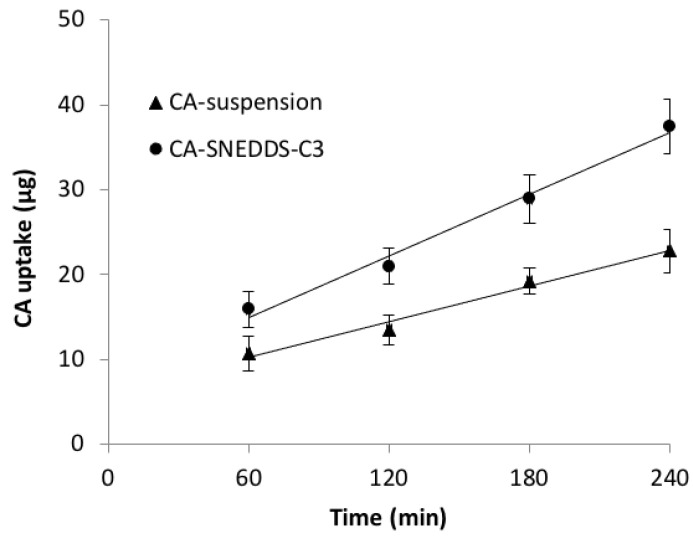
CA uptake in Caco-2 cells after 0.5 mM CA was applied (*n* = 3; mean ± SD).

The current study shows that the CA can accumulate in Caco-2 cell monolayers intact, indicating that it can enter systemic circulation and be hydrolyzed by blood-borne esterases and/or metabolized by the liver. As shown in Figure 6, the CA-SNEDDS-C3 cellular uptake was greater than that of the free CA (CA suspension). The HPLC results are shown in supplementary Tables S1 and S2. This could be due to the increased lipid solubility at the absorption site, a faster rate of diffusion through cells, and/or P-gp inhibition by PEG-400 in CA-SNEDDS-C3.

## 4. Conclusions

In conclusion, CA-loaded liquid SNEDDS were successfully developed using the aqueous titration method. The obtained results showed that the prepared SNEDDS was thermodynamically stable, with good self-emulsification efficiency and a globule size in the nanometric range, indicating that it may be physiologically stable. The selected CA-SNEDDS-C3 showed a higher dissolution rate than free CA (CA suspension). Human intestinal Caco-2 cells uptake CA much better in SNEDDS than in suspension, indicating that the lipid solubility of CA is higher and the rate of diffusion through cells is faster when it is formulated in SNEDDS. Our findings suggest that CA-SNEDDS could be considered a potential alternative for oral delivery. To summarize, it is a novel and commercially feasible alternative to the currently marketed CA products.

## Figures and Tables

**Table 1:** Formulation components of the SNEDDS formulations of CA (C1–C10) prepared using castor oil, cremophor EL, PEG400 and water.

Formulation Code	Castor Oil (% *v*/*v*)	Smix (Cremophor EL:PEG 400; 2:1) (% *v*/*v*)	Water (% *v*/*v*)
C1	5	35	60
C2	5	40	55
C3	5	45	50
C4	5	30	65
C5	7.84	47.06	45.1
C6	8.13	56.91	34.96
C7	11	54.14	34.86
C8	11.95	57.36	30.7
C9	13.79	55.17	31.03
C10	14.81	59.26	25.93

**Table 2:** An overview of the findings from the constructed pseudo-ternary phase diagram.

Figures	Smix Ratio	Self-Nanoemulsifying Area	Maximum Oil Phase Emulsified (% *v*/*v*)	Maximum Amount of Smix Used to Emulsify Respective Maximum Oil Phase (% *v*/*v*)
Figure 2A	1:0	Lowest	10	80
Figure 2B	1:1	Higher than 2A	15.44	74.51
Figure 2C	1:2	Lower than 2A	11.24	78.65
Figure 2D	2:1	Highest	17.21	72.36
Figure 2E	3:1	Lower than 2D	12.82	76.92
Figure 2F	4:1	Lower than 2D and 2E	8.13	56.91

**Table 3:** Thermodynamic stability and self-nano-emulsification testing results in the presence of deionized water, 0.1 N HCl, and phosphate buffer (pH 6.8).

Formulation Code	Emulsification Time (s)	Grade of Self-Nanoemulsification Test	Thermodynamic Stability Tests		
			Centrifugation	Heating and Cooling Cycles	Freeze-Thaw Cycles
C1	16.67 ± 0.59	A	✓	✓	✓
C2	17.93 ± 0.61	A	✓	✓	✓
C3	15.79 ± 0.40	A	✓	✓	✓
C4	20.67 ± 0.62	A	✓	✓	✓
C5	16.75 ± 0.84	A	✓	✓	✓
C6	20.82 ± 0.54	A	✓	✓	✓
C7	22.06 ± 0.64	A	✓	✓	✓
C8	20.07 ± 0.63	A	✓	✓	✓
C9	20.75 ± 0.51	A	✓	✓	✓
C10	21.42 ± 0.68	A	✓	✓	✓

**Table 4:** Characterization of the CA-loaded SNEDDS. Mean ± SD, *n* = 3.

Formulation Code	Mean Droplet Size (nm)	Poly Dispersity Index (PDI)	Zeta Potential (mV)	Transmittance (%)	Drug Loading Efficiency (%)
C1	23.33± 1.07	0.116 ± 0.008	−19.71 ± 1.05	97.41 ± 0.34	96.44 ± 1.33
C2	21.23± 1.90	0.084 ± 0.009	−21.53 ± 1.64	98.23 ± 0.15	97.07 ± 1.47
C3	18.50± 1.83	0.064 ± 0.008	−22.12 ± 1.20	98.32 ± 0.27	97.62 ± 1.06
C4	26.17± 2.05	0.132 ± 0.011	−18.88 ± 1.78	96.42 ± 0.25	96.25 ± 1.22
C5	23.80± 1.45	0.106 ± 0.005	−19.91 ± 1.37	97.23 ± 0.06	98.76 ± 0.68
C6	25.98± 1.48	0.129 ± 0.009	−17.78 ± 1.52	96.43 ± 0.31	98.17 ± 0.83
C7	32.07± 1.11	0.168 ± 0.018	−12.35 ± 2.06	96.23 ± 0.15	99.41 ± 0.22
C8	33.43± 0.83	0.170 ± 0.020	−12.77 ± 1.79	96.47 ± 0.31	99.16 ± 0.48
C9	27.33± 1.70	0.140 ± 0.014	−14.36 ± 1.89	96.32 ± 0.26	99.04 ± 0.23
C10	27.97± 2.32	0.156 ± 0.010	−15.47 ± 1.75	96.31 ± 0.20	98.93 ± 0.55

## Data Availability

The data presented in this study is available in the article.

## Supplementary Materials

The following supporting information can be downloaded at: https://www.mdpi.com/article/10.3390/pharmaceutics14040772/s1, Table S1: The HPLC results of CA uptake in Caco-2 cells after 0.5 mM CA was applied (*n* = 3; mean ± SD); Table S2: CA concentration (ug/mL) analyzed from HPLC data after cellular uptake (*n* = 3; mean ± SD).
