# Supplementary material for: Optimization of a Cefuroxime Axetil-Loaded Liquid Self-Nanoemulsifying Drug Delivery System: Enhanced Solubility, Dissolution and Caco-2 Cell Uptake
Source: Pharmaceutics. 2022 Apr 1;14(4):772. doi: 10.3390/pharmaceutics14040772 (PMC9028143; doi:10.3390/pharmaceutics14040772)
Supplement: Supplementary file 1 [file pharmaceutics-14-00772-s001.zip › pharmaceutics-1646830-supplementary.pdf]

Supplementary Material

Optimization of a Cefuroxime Axetil-Loaded Liquid Self-Nanoemulsifying Drug Delivery System: Enhanced Solubility, Dissolution and Caco-2 Cell Uptake

Arshad Ali Khan, Akhtar Atiya, Safia Akhtar, Yogesh Yadav, Kamal A. Qureshi, Mariusz Jaremko and Syed Mahmood

Table S1. The HPLC results of CA uptake in Caco-2 cells after 0.5 mM CA was applied ($n = 3$; mean ± SD).

**CA-suspension**		**HPLC Peak Area**		
	**Time (min)**	**Sample 1**	**Sample 2**	**Sample 3**
	60	1988	2512	2900
	120	2694	3150	3469
	180	4108	4313	4791
	240	4586	5361	5748
**CA-SNEDDS-C3**		**HPLC Peak Area**		
	**Time (min)**	**Sample 1**	**Sample 2**	**Sample 3**
	60	3150	3766	4062
	120	4290	4928	5224
	180	6728	7230	5931
	240	7708	8848	9122

Table S2. CA concentration (ug/ml) analyzed from HPLC data after cellular uptake ($n = 3$; mean ± SD).

**CA-suspension**		**CA (µg/mL)**				
	**Time (min)**	**Sample 1**	**Sample 2**	**Sample 3**	**Avg**	**STD**
	60	8.6	10.9	12.6	10.70	2.01
	120	11.7	13.7	15.1	13.50	1.71
	180	17.9	18.8	20.9	19.20	1.54
	240	20	23.4	25.1	22.83	2.60
**CA-SNEDDS-C3**		**CA (µg/mL)**				
	**Time (min)**	**Sample 1**	**Sample 2**	**Sample 3**	**Avg**	**STD**
	60	13.7	16.4	17.7	15.93	2.04
	120	18.7	21.5	22.8	21.00	2.10
	180	29.4	31.6	25.9	28.97	2.87
	240	33.7	38.7	39.9	37.43	3.29
